# Molecular Surveillance of Antimalarial Drug Resistance-Associated Mutations in *P. falciparum* Imported from Africa into Guangdong Province, China, 2023–2025

**DOI:** 10.3390/pathogens15101080

**Published:** 2026-10-14

**Authors:** Lijun Chen, Qiang Mao, De Wu, Jiahui Liu, Jiayi Zhang, Wencheng Lu, Minwu Peng, Yating Chen, Jieling Wang, Jingdiao Chen, Mang Shi

**Affiliations:** 1 Parasitic Disease Prevention and Control Institute, Guangdong Center for Disease Control and Prevention, Guangzhou 511430, China13450381543@163.com (Q.M.); 18306616472@163.com (J.L.); wangjl7.thaa@vip.163.com (J.W.);; 2 State Key Laboratory for Biocontrol, School of Medicine, Shenzhen Campus, Sun Yat-sen University, Shenzhen 518107, China; 3 Shenzhen Key Laboratory for Systems Medicine in Inflammatory Diseases, Shenzhen Campus, Sun Yat-sen University, Shenzhen 518107, China; 4 National Key Laboratory of Intelligent Tracking and Forecasting for Infectious Diseases, School of Medicine, Shenzhen Campus, Sun Yat-sen University, Shenzhen 518107, China

**Keywords:** *Plasmodium falciparum*, *pfk13*, *pfcrt*, *pfmdr1*, drug resistance, Africa, China

## Abstract

Imported falciparum malaria remains an important challenge in China, particularly with the emergence of antimalarial resistance in Africa. This study characterized resistance-associated mutations in *pfk13*, *pfcrt* and *pfmdr1* by nested PCR and Sanger sequencing of 291 *Plasmodium falciparum* (*P. falciparum*) isolates imported from 29 African countries into Guangdong Province during 2023–2025. Sequences were obtained for *pfk13* (*n* = 279), *pfcrt* (*n* = 225) and *pfmdr1* (*n* = 235). Four *pfk13* variants—C469Y, Y558C, R561H and A675V—were each detected once (0.36%). These isolates originated from East and Central Africa. The wild-type *pfk13* haplotype accounted for 98.57% of isolates. For *pfcrt*, the wild-type MNK haplotype predominated (85.33%), whereas the IET triple-mutant haplotype accounted for 14.22% and was mainly detected in isolates from West and Central Africa. Y184F was the predominant *pfmdr1* variant (55.32%), with an annual prevalence of 52.59–60.78%. Other variants included N86Y (3.40%), D1246Y (1.28%), and S163N, T199S and S1217F (0.43% each). The Y184F single-mutant haplotype was most common (51.91%). The imported isolates showed a low prevalence of *pfk13* variants, predominance of wild-type *pfcrt* and frequent *pfmdr1* Y184F variants. The detection of several *pfk13* variants highlights the need for continued molecular surveillance of antimalarial resistance.

## 1. Introduction

Malaria is a mosquito-borne parasitic disease caused by *Plasmodium* spp. In 2024, malaria remained endemic in 80 countries and territories worldwide, with an estimated 282 million cases and 610,000 deaths [1]. The African region bore the greatest burden, accounting for approximately 94% of global cases (265 million) and 95% of malaria-related deaths (approximately 579,000) [1]. China has reported no indigenous malaria cases since 2017 and was certified malaria-free by the World Health Organization (WHO) in 2021 [2]. Nevertheless, Guangdong Province maintains extensive economic ties and frequent population movement with other countries. The large number of residents travelling to malaria-endemic regions, particularly Africa and Southeast Asia, for work, business and tourism creates sustained pressure from imported malaria and a potential risk of re-establishing local transmission.

The number of imported malaria cases in Guangdong Province has increased in recent years, with most infections acquired in Africa and *Plasmodium falciparum* (*P. falciparum*) being the predominant species [3]. *Falciparum malaria* can progress rapidly and may lead to severe disease or death if not promptly diagnosed and treated. Artemisinin-based combination therapies (ACTs) remain the cornerstone of *falciparum malaria* treatment. However, prolonged and widespread antimalarial use has imposed strong selective pressure, facilitating the emergence and spread of drug-resistant parasites [4,5,6]. In 2020, an indigenous artemisinin-resistant *P. falciparum* lineage was first reported in Africa [7]. Given that most imported malaria cases in Guangdong originate from African countries, surveillance of antimalarial resistance among these infections is essential for informing case management and strengthening malaria prevention and control.

Three resistance-associated genes have been identified in previous studies: *P. falciparum* kelch 13 gene (*pfk13*), the chloroquine resistance transporter gene (*pfcrt*) and multidrug resistance gene 1 (*pfmdr1*). Variants in the propeller domain of *pfk13* are established molecular markers of partial artemisinin resistance and are widely used to monitor changes in artemisinin susceptibility [8,9]. Variants in *pfcrt* are associated with altered susceptibility to chloroquine and several ACT partner drugs, whereas *pfmdr1* polymorphisms can modulate parasite susceptibility to partner drugs such as amodiaquine and lumefantrine [10]. Combined surveillance of these genes provides a broader molecular profile of parasite susceptibility to artemisinin and its partner drugs.

In this study, we investigated molecular markers of antimalarial resistance in *P. falciparum* isolates imported from Africa into Guangdong Province between 2023 and 2025. Our study aimed to characterize the prevalence and geographical distribution of these molecular markers, thereby providing evidence to support the surveillance and management of imported *falciparum malaria* in Guangdong Province and elsewhere in China. To our knowledge, this is the first province-wide molecular surveillance of these resistance-associated variants in Guangdong Province.

## 2. Materials and Methods

### 2.1. Sampling

This retrospective study analyzed isolates obtained from the DNA biobank of Africa-imported *P. falciparum* isolates in Guangdong Province between 2023 and 2025, which had been stored at −20 °C in our laboratory. All isolates were collected before antimalarial drug administration. Genomic DNA was extracted from 200 µL of EDTA-anticoagulated whole blood using a magnetic bead-based nucleic acid extraction and purification kit. Species identification was initially performed by microscopy, and single-species *P. falciparum* infection was further confirmed by species-specific real-time PCR; mixed-species infections were excluded. Parasitaemia was not quantified by microscopy in this study; instead, parasite DNA load was assessed by real-time PCR, and Ct values were recorded for each sample as an indicator of relative parasite density.

### 2.2. Reagents and Instruments

#### 2.2.1. Reagents

Platinum™ SuperFi II Green PCR Master Mix (Invitrogen, Waltham, MA, USA); agarose (VETEC, Sigma-Aldrich, St. Louis, MO, USA); GelGreen^®^ (Biotium, Fremont, CA, USA).

#### 2.2.2. Instruments

PCR thermal cycler (Veriti DX 96, Applied Biosystems, Waltham, MA, USA); electrophoresis system (Mupid-2plus, AD110, Mupid Co., Ltd., Japan); gel imaging system (Bio-Rad Gel Doc XR+, Bio-Rad Laboratories, Hercules, CA, USA).

### 2.3. Nested PCR Amplification

Target regions of *pfk13*, *pfcrt* and *pfmdr1* were amplified using nested PCR. The first-round PCR was performed in a total volume of 10 μL containing 5 μL of 2× PCR Master Mix, 0.3 μL each of the forward and reverse outer primers (10 μM), 1 μL of DNA template and nuclease-free water. The second-round PCR was performed in a total volume of 30 μL containing 15 μL of 2× PCR Master Mix, 0.9 μL each of the forward and reverse inner primers (10 μM), 1.5 μL of the first-round PCR product and nuclease-free water. Negative controls (nuclease-free water instead of DNA template) were included in each PCR run and loaded on the same agarose gel as the test samples; no amplification was observed in any negative control. Subsequently, 10 μL of each PCR product was separated on a 1.0% agarose gel stained with GelGreen and visualized under ultraviolet illumination.

#### 2.3.1. Amplification of *pfk13*

The first-round primers, *pfk13*-1F (5′-CGGAGTGACCAAATCTGGGA-3′) and *pfk13*-1R (5′-GGGAATCTGGTGGTAACAGC-3′), generated a 2097-bp amplicon. The second-round primers, *pfk13*-2F (5′-CAAGCTGCCATTCATTTG-3′) and *pfk13*-2R (5′-GCCTTGTTGAAAGAAGCAGAGC-3′), generated an 849-bp amplicon. PCR cycling conditions consisted of an initial denaturation at 95 °C for 2 min, followed by 30 cycles of denaturation at 95 °C for 30 s, annealing at 60 °C for 90 s and extension at 72 °C for 90 s, with a final extension at 72 °C for 10 min [11]. The primers were designed to amplify codons 427–709 of pfk13. After quality trimming, the final analyzed sequences covered codons 447–676, which cover part of the propeller (Kelch-repeat) domain and contain all WHO-validated resistance-associated codons except F446I (codon 446) [12]. The BTB/POZ domain was not covered, as the analyzed region begins at codon 447.

#### 2.3.2. Amplification of *pfcrt*

The first-round primers, *pfcrt*-1F (5′-CCCTTGTCGACCTTAACAGATG-3′) and *pfcrt*-1R (5′-AAAATGACTGAACAGGCATCTAAC-3′), generated a 534-bp amplicon. The second-round primers, *pfcrt*-2F (5′-TCTTGGTAAATGTGCTCATGTG-3′) and *pfcrt*-2R (5′-AAAGTTGTGAGTTTCGGATGTT-3′), generated a 178-bp amplicon. PCR cycling conditions consisted of an initial denaturation at 95 °C for 15 min, followed by 30 cycles of denaturation at 95 °C for 30 s, annealing at 58 °C for 30 s and extension at 72 °C for 30 s, with a final extension at 72 °C for 5 min [13]. The primers were designed to amplify codons 41–99 of *pfcrt.* After quality trimming, the final analyzed sequences covered codons 66–86, which include the key chloroquine resistance codons 72–76 [12].

#### 2.3.3. Amplification of *pfmdr1*

Two sets of nested primers, designated a and b, were used to amplify the upstream and downstream regions of *pfmdr1*, respectively. For the upstream region, the first-round primers *pfmdr1*-a1F (5′-TTAAATGTTTACCTGCACAACATAGAAAATT-3′) and *pfmdr1*-a1R (5′-CTCCACAATAACTTGCAACAGTTCTTA-3′) generated a 611-bp amplicon. The second-round primers *pfmdr1*-a2F (5′-TGTATGTGCTGTATTATCAGGA-3′) and *pfmdr1*-a2R (5′-CTCTTCTATAATGGACATGGTA-3′) generated a 526-bp amplicon. For the downstream region, the first-round primers *pfmdr1*-b1F (5′-AATTTGATAGAAAAAGCTATTGATTATAA-3′) and *pfmdr1*-b1R (5′-TATTTGGTAATGATTCGATAAATTCATC-3′) generated an 877-bp amplicon. The second-round primers *pfmdr1*-b2F (5′-GAATTATTGTAAATGCAGCTTTA-3′) and *pfmdr1*-b2R (5′-GCAGCAAACTTACTAACACG-3′) generated a 799-bp amplicon. For both regions, the first-round PCR conditions consisted of an initial denaturation at 95 °C for 3 min, followed by 35 cycles of denaturation at 93 °C for 30 s, annealing at 52 °C for 30 s and extension at 72 °C for 30 s, with a final extension at 72 °C for 5 min. The same cycling conditions were used for the second-round PCR [11]. The upstream primers were designed to amplify codons 62–236 of *pfmdr1*, and the downstream primers were designed to amplify codons 1024–1288. After quality trimming, the final analyzed sequences covered codons 86–228 for the upstream region and codons 1052–1271 for the downstream region, which together include the key codons 86, 184 and 1246 [14].

### 2.4. DNA Sequencing and Sequence Analysis

PCR products were subjected to bidirectional Sanger sequencing by Sangon Biotech (Shanghai) Co., Ltd., Shanghai, China. Reference sequences for *P. falciparum pfk13* (NC_004331.3), *pfcrt* (NC_004328.3) and *pfmdr1* (NC_004326.2) were retrieved from the National Center for Biotechnology Information database. Sequences were aligned with their respective reference sequences using MEGA version 5.0 to identify nucleotide substitutions, amino acid changes and haplotypes. Chromatograms were manually inspected. Positions with a clear dominant peak were scored as the consensus base. Positions showing ambiguous or mixed signals were re-checked manually, and the base with higher signal intensity was scored as the consensus base; however, low-level minority variants may have been missed.

### 2.5. Statistical Analysis

Statistical analyses were performed using SPSS version 25.0. Categorical variables are presented as numbers and percentages. No formal statistical comparisons were performed due to the small number of variant-carrying isolates.

## 3. Results

### 3.1. Characteristics of the Study Samples

A total of 291 isolates of *P. falciparum* imported from 29 African countries were included in this study. Among them, 136 samples were collected in 2023, 85 in 2024, and 70 in 2025 respectively (Table 1). The six most frequently reported countries of origin were Nigeria (*n* = 55), the Democratic Republic of the Congo (*n* = 43), Cameroon (*n* = 21), Tanzania (*n* = 20), Guinea (*n* = 18) and Uganda (*n* = 18).

### 3.2. Variant Profiles of Resistance-Associated Genes

Target regions of *pfk13*, *pfcrt* and *pfmdr1* were amplified from the 291 samples by nested PCR. A total of 1164 amplicons were submitted for sequencing, of which 974 were successfully sequenced. These comprised 279 *pfk13* sequences, 225 *pfcrt* sequences and 470 *pfmdr1* sequences representing two target regions from 235 samples.

Among the 279 *pfk13* sequences, nucleotide substitutions were detected in nine isolates. Four isolates carried nonsynonymous variants—C469Y, Y558C, R561H and A675V—whereas the remaining five carried synonymous variants (Table S1). Each nonsynonymous variant was detected in one isolate, corresponding to a frequency of 0.36% (1/279) (Table 2). Among the 225 *pfcrt* sequences, 33 isolates carried nonsynonymous variants at three codons: M74I, N75E and K76T, whereas no synonymous variants were detected. The frequencies of these three variant sites were 14.22%, 14.67% and 14.67% (Table 2). Nucleotide substitutions were detected in 148 of the 235 *pfmdr1* sequences, comprising six nonsynonymous and six synonymous variants (Table S1), with missense variants present in 133 isolates. Y184F was the most frequent nonsynonymous variant (55.32%), followed by N86Y (3.40%) and D1246Y (1.28%). S163N, T199S and S1217F were each detected in one isolate (0.43%) (Table 2).

### 3.3. Geographical and Annual Distribution of Variant-Carrying Isolates

The four isolates carrying nonsynonymous *pfk13* variants originated from East and Central Africa. C469Y, Y558C, R561H and A675V were detected in isolates imported from Uganda, the Democratic Republic of the Congo, Rwanda and Kenya, respectively. Isolates carrying the *pfcrt* M74I, N75E and K76T variants were mainly imported from Central and West Africa. These variants were frequently detected among isolates originating from Nigeria, Liberia and Guinea. The *pfmdr1* Y184F variant was widely distributed among isolates imported from multiple malaria-endemic African countries. All three isolates carrying D1246Y originated from the Comoros (Table 2).

No nonsynonymous *pfk13* variants were detected in 2023. One A675V variant was detected in 2024, corresponding to a frequency of 1.19%. Three variants—C469Y, Y558C and R561H—were detected in 2025, each with a frequency of 1.49%. The *pfcrt* M74I, N75E and K76T variants were detected at frequencies of 10.94–16.38%. The *pfmdr1* Y184F variant was detected at frequencies of 52.59–60.78%. N86Y and D1246Y occurred at relatively low frequencies of 0–5.17%, whereas the remaining variants were detected only sporadically (Table 3).

### 3.4. Haplotype Distribution

The wild-type *pfk13* haplotype, C_469_Y_558_R_561_A_675_, predominated and accounted for 98.57% of the isolates. Four single-mutant haplotypes were detected, each in one isolate (0.36%). For *pfcrt*, the wild-type MNK haplotype (M_74_N_75_K_76_) was the most prevalent, accounting for 85.33% of the isolates. The triple-mutant IET haplotype (I_74_E_75_T_76_) accounted for 14.22%, whereas the double-mutant MET haplotype (M_74_E_75_T_76_) was detected in one isolate (0.44%). Eight *pfmdr1* haplotypes were identified. The wild-type haplotype and the Y184F single-mutant haplotype accounted for 43.40% and 51.91% of the isolates, respectively. Each of the remaining single-, double- and triple-mutant haplotypes accounted for less than 2% of the isolates (Table 4).

## 4. Discussion

This study characterized variants in three antimalarial resistance-associated genes among *P. falciparum* isolates imported from Africa into Guangdong Province. *pfk13* variants were infrequent but occurred at multiple codons, including validated markers of artemisinin partial resistance. The wild-type *pfcrt* haplotype predominated, whereas the classical CVIET variant haplotype occurred at a relatively low frequency. In contrast, *pfmdr1* was characterized by a high prevalence of Y184F and several uncommon haplotypes. These findings provide a contemporary overview of antimalarial resistance-associated markers among imported cases and support the need for continued molecular surveillance. However, in the absence of phenotypic susceptibility and clinical outcome data, and given the uneven sampling across source countries, these findings should not be interpreted as direct evidence of clinical resistance or as population-level prevalence estimates for the corresponding African countries.

Variants in the propeller domain of *pfk13* are established molecular markers of artemisinin partial resistance [15]. Such variants were initially identified in Southeast Asia and subsequently spread across the Greater Mekong Subregion [16], while independently emerging lineages have more recently been reported in Africa. C469Y, R561H and A675V are classified by WHO as validated markers of artemisinin partial resistance [12]. In particular, R561H underwent clonal expansion in Rwanda and was associated with delayed parasite clearance [7], and several other variants, including C469Y and A675V, have emerged in different African regions [9,17]. In the present study, only four of the 279 successfully sequenced isolates carried nonsynonymous *pfk13* variants. R561H was detected in an isolate imported from Rwanda, consistent with its previously reported emergence in that country [7], while C469Y was identified in an isolate from Uganda, where its indigenous emergence and spread have recently been demonstrated [18]. A675V, another validated marker of artemisinin partial resistance, was detected in an isolate from Kenya. By contrast, the functional significance of Y558C, detected in an isolate from the Democratic Republic of the Congo, remains unclear [17]. Y558C is currently classified by WHO as a novel and unvalidated variant because it has not been associated with delayed parasite clearance [12]. No C580Y variant, which is widespread in Southeast Asia [15], was detected. It is worth noting that no R622I variant was found, most likely due to the underrepresentation of travellers from the Horn of Africa in this sample. The R622I variant has been primarily reported in the Horn of Africa, particularly in Eritrea, where its prevalence increased from 8.6% in 2016 to 21.0% in 2019 [19]. Overall, the occurrence of multiple low-frequency *pfk13* variants was broadly consistent with previous studies of African isolates imported into China [11,20,21] and did not indicate the predominance of a single resistance-associated lineage. Although three of the four variants were detected in 2025, the small number of variant-carrying isolates and differences in annual sample sizes preclude any conclusion regarding an increasing temporal trend.

We also find the predominance of the wild-type *pfcrt* CVMNK haplotype. Both CVMNK and CVIET were assigned based on directly sequenced codons 72–76, which were fully covered by our final analyzed sequences (codons 66–86). Chloroquine was used extensively following its introduction in the 1930s [22], and chloroquine-resistant *P. falciparum* subsequently emerged and spread worldwide [23]. Variants in *pfcrt*, particularly K76T, were later established as major determinants of chloroquine resistance through their effects on drug transport across the digestive vacuole membrane [24]. The CVIET haplotype, which became the predominant chloroquine-resistant haplotype in Africa, is considered to have originated in Southeast Asia before spreading into Africa [25]. In this study, CVIET accounted for 14.22% of the isolates, lower than the 16.67% reported among African isolates imported into Jiangxi Province during 2017–2020 and the 29.8% reported in a nationwide study during 2012–2015 [11,26]. Conversely, the wild-type CVMNK haplotype accounted for 85.33%. This may reflect not only the re-expansion of wild-type *pfcrt* genotypes following the withdrawal of chloroquine and the resulting reduction in drug-selection pressure, but also ongoing selection associated with artemether–lumefantrine use, which has been shown to actively select parasites carrying the wild-type K76 allele [27]. Differences between studies may also reflect variation in sampling periods and the geographical composition of imported cases. Importantly, the predominance of the wild-type genotype should not be interpreted as direct evidence of restored clinical susceptibility to chloroquine.

We further examined *pfmdr1*, which encodes a digestive vacuole membrane transporter that can modulate susceptibility to several antimalarial drugs, often in interaction with *pfcrt* [14]. Among the variants detected, Y184F, N86Y and D1246Y are well-characterized resistance-associated variants [14], whereas S163N, T199S and S1217F are low-frequency variants with uncertain clinical relevance [28]. Y184F was the predominant variant, with an overall frequency of 55.32%, whereas N86Y occurred at a substantially lower frequency of 3.40%. The Y184F frequency was higher than the 24.7% reported in a previous nationwide study but lower than the 78% reported among African isolates imported into Jiangsu Province between 2011 and 2019 [20]. These differences may reflect variation in sampling periods, countries of infection or antimalarial selection pressures. In this context, it is relevant that first-line ACT regimens vary across African countries. According to the World Malaria Report 2025, artemether–lumefantrine (AL) is the most widely used first-line ACT in Africa, while artesunate–amodiaquine (AS-AQ) and dihydroartemisinin–piperaquine (DHA-PPQ) are also used as first-line or alternative regimens in some countries [1]. Artemether–lumefantrine has been reported to preferentially select parasites carrying the N86 and 184F alleles, whereas dihydroartemisinin–piperaquine may exert selection in the opposite direction [29]. This N86 selection by AL has been demonstrated in vivo [30]. Genome-editing studies show that N86Y increases resistance to amodiaquine and chloroquine but increases susceptibility to lumefantrine, mefloquine and dihydroartemisinin, whereas Y184F reduces piperaquine susceptibility in the N86 background [31]. The N86/184F haplotype has also been selected in Southeast Asia with *pfmdr1* amplification, conferring increased transporter activity and reduced lumefantrine susceptibility [32]. The high frequency of Y184F, low frequency of N86Y and predominance of the N86/184F haplotype observed here are compatible with the molecular pattern associated with artemether–lumefantrine selection. Nevertheless, a causal relationship cannot be established without detailed information on previous drug exposure and treatment practices in the countries of infection.

Several uncommon *pfmdr1* haplotypes were also detected. All three D1246Y variants occurred within the YFY haplotype (86Y/184F/1246Y), and all three isolates originated from the Comoros. This haplotype has previously been reported in Tanzania, with marked geographical enrichment in the Tanga region [33], suggesting that uncommon *pfmdr1* haplotypes may exhibit geographical clustering. T199S, previously reported as a low-frequency emerging variant in western Kenya [28], was also detected, together with two poorly characterized substitutions, S163N and S1217F, both of which occurred in combination with Y184F. These rare polymorphisms expand the observed diversity of *pfmdr1* but require confirmation in independent datasets and functional studies before they can be considered resistance markers.

This study has several limitations. First, the number of cases differed substantially among countries, and several countries were represented by only a few successfully sequenced isolates, which may limit the generalizability of our findings. In addition, given the small number of variant-carrying isolates and unequal sample sizes across years, no temporal conclusion can be drawn from these data. Second, sequencing success also varied among the three genes, potentially introducing detection bias. Third, this study focused on three genes, which may limit the resolution of the resistance landscape; future studies should extend the panel to include additional resistance-associated loci such as *dhps*/*dhfr*, *aat1*, *ubp1*, *pfcarl*, and *px1* [34,35,36,37,38]. Fourth, multiclonal infections were not systematically assessed using dedicated genotyping markers such as *msp1*, *msp2*, glurp, *Poly-α*, *PfPK2* or *TA1* [39,40], which may lead to underdetection of minority clones. This limitation is further compounded by the limited sensitivity of targeted Sanger sequencing for minority variants in mixed infections, which also does not capture broader parasite genomic diversity or assess *pfmdr1* copy-number variation and other resistance-associated loci. Finally, no in vitro susceptibility testing or clinical treatment-response data were available; therefore, associations between the detected genotypes and phenotypic or clinical resistance could not be evaluated directly. The biological significance of several uncommon variants also remains uncertain.

Based on our findings, we propose the following recommendations. For clinical practice, continued molecular surveillance of *pfk13*, *pfcrt* and *pfmdr1* in imported malaria cases is needed to inform case management and detect emerging resistance early. For research, future studies should expand the gene panel to include additional resistance-associated loci, incorporate clonality markers, and integrate in vitro susceptibility testing and clinical treatment-response data. For public health, strengthening surveillance of imported malaria and maintaining vigilance for *pfk13* variants, particularly those emerging in Africa, is essential for preventing the re-establishment of local transmission in Guangdong Province.

## Figures and Tables

**Table 1:** Country distribution of *Plasmodium falciparum* *(P. falciparum*) isolates imported from Africa.

Country of Origin	2023	2024	2025	Total	Proportion (%)
Nigeria	33	14	8	55	18.90
Democratic Republic of the Congo	20	9	14	43	14.78
Cameroon	7	9	5	21	7.22
Tanzania	5	7	8	20	6.87
Guinea	10	4	4	18	6.19
Uganda	9	5	4	18	6.19
Côte d’Ivoire	9	4	3	16	5.50
Ghana	6	3	6	15	5.15
Angola	3	6	5	14	4.81
Sierra Leone	8	2	1	11	3.78
Liberia	4	5	1	10	3.44
Mali	2	4	1	7	2.41
Republic of the Congo	3	2	1	6	2.06
Comoros	3	3	0	6	2.06
Burkina Faso	3	0	1	4	1.37
Kenya	1	3	0	4	1.37
Mozambique	1	0	3	4	1.37
Zambia	1	0	2	3	1.03
Ethiopia	1	1	0	2	0.69
Togo	1	0	1	2	0.69
Gabon	1	1	0	2	0.69
Niger	2	0	0	2	0.69
Chad	2	0	0	2	0.69
Benin	0	1	0	1	0.34
Equatorial Guinea	0	1	0	1	0.34
Zimbabwe	1	0	0	1	0.34
Rwanda	0	0	1	1	0.34
Namibia	0	1	0	1	0.34
Senegal	0	0	1	1	0.34
Total	136	85	70	291	100

Notes: ^1^ Data are presented as numbers (n) and proportions (%). A total of 291 isolates from 29 African countries were analyzed. ^2^ Percentages may not sum to 100.00 due to rounding.

**Table 2:** Distribution of *pfk13*, *pfcrt* and *pfmdr1* variants in successfully sequenced isolates from different countries and geographic regions of Africa.

African Sub- Region	Source Countries	Successfully Sequenced Isolates for *pfk13*	*pfk13* Variant Sites (No. of Isolates, Variant Frequency, %)	Successfully Sequenced Isolates for *pfcrt*	*pfcrt* Variant Sites (No. of Isolates, Variant Frequency, %)	Successfully Sequenced Isolates for *pfmdr1*	*pfmdr1* Variant Sites (No. of Isolates, Variant Frequency, %)
East Africa	Tanzania	18	-	15	-	18	Y184F (9, 50.00)
	Uganda	17	C469Y (1, 5.88)	13	-	16	Y184F (6, 37.50), T199S (1, 6.25)
	Comoros	6	-	4	-	4	N86Y (4, 100), Y184F (3, 75.00), D1246Y (3, 75.00)
	Kenya	4	A675V (1, 25.00)	3	-	3	Y184F (3, 100)
	Ethiopia	2	-	1	-	2	Y184F (2, 100)
	Rwanda	1	R561H (1, 100)	1	-	1	Y184F (1, 100)
Southern Africa	Angola	13	-	11	-	12	Y184F (2, 16.67)
	Mozambique	3	-	4	-	3	Y184F (2, 66.67)
	Zambia	3	-	2	-	3	-
	Namibia	1	-	1	-	1	Y184F (1, 100)
	Zimbabwe	1	-	1	-	1	-
Central Africa	Democratic Republic of the Congo	42	Y558C (1, 2.38)	34	M74I (1, 2.94), N75E (1, 2.94), K76T (1, 2.94)	35	Y184F (15, 42.86)
	Cameroon	20	-	15	-	16	Y184F (7, 43.75), N86Y (1, 6.25)
	Republic of the Congo	6	-	5	M74I (2, 40.00), N75E (2, 40.00), K76T (2, 40.00)	4	Y184F (2, 50.00)
	Gabon	2	-	2	M74I (1, 50.00), N75E (1, 50.00), K76T (1, 50.00)	2	Y184F (1, 50.00)
	Equatorial Guinea	1	-	1	-	0	-
	Chad	2	-	2	M74I (1, 50.00), N75E (1, 50.00), K76T (1, 50.00)	2	Y184F (1, 50.00)
West Africa	Nigeria	53	-	46	M74I (9, 19.57), N75E (9, 19.57), K76T (9, 19.57)	40	Y184F (28, 70.00), S1217F (1, 2.50)
	Guinea	17	-	15	M74I (8, 53.33), N75E (8, 53.33), K76T (8, 53.33)	15	Y184F (8, 53.33)
	Côte d’Ivoire	15	-	11	M74I (1, 9.09), N75E (2, 18.18), K76T (2, 18.18)	13	Y184F (8, 61.54)
	Ghana	15	-	10	M74I (2, 20.00), N75E (2, 20.00), K76T (2, 20.00)	14	Y184F (10, 71.43)
	Sierra Leone	11	-	9	M74I (1, 11.11), N75E (1, 11.11), K76T (1, 11.11)	11	Y184F (8, 72.73), S163N (1, 9.09)
	Liberia	10	-	8	M74I (5, 62.50), N75E (5, 62.50), K76T (5, 62.50)	5	Y184F (1, 20.00)
	Burkina Faso	4	-	3	-	4	Y184F (3, 75.00)
	Mali	6	-	3	-	5	Y184F (4, 80.00), N86Y (1, 20.00)
	Togo	2	-	2	M74I (1, 50.00), N75E (1, 50.00), K76T (1, 50.00)	2	Y184F (2, 100)
	Niger	2	-	2	-	2	N86Y (2, 100), Y184F (2, 100)
	Benin	1	-	1	-	0	-
	Senegal	1	-	0	-	1	Y184F (1, 100)
Total		279	C469Y (1, 0.36), Y558C (1, 0.36), R561H (1, 0.36), A675V (1, 0.36)	225	M74I (32, 14.22), N75E (33, 14.67), K76T (33, 14.67)	235	N86Y (8, 3.40), S163N (1, 0.43), Y184F (130, 55.32), T199S (1, 0.43), S1217F (1, 0.43), D1246Y (3, 1.28)

Notes: ^1^ Variant frequency = (Variant-carrying isolates/Successfully sequenced isolates of the country) × 100%, rounded to two decimal places. ^2^ “-” means no variant was identified for this gene, or data unavailable with zero successfully sequenced isolates.

**Table 3:** Annual distribution of variant frequencies for each missense variant in *pfk13*, *pfcrt*, and *pfmdr1* of *P. falciparum* isolates imported from Africa.

Gene	Nucleotide Mutation	Amino Acid Substitution	Variant-Carrying Isolates/Total Isolates (Variant Frequencies, %)		
			2023	2024	2025
*pfk13* (*n* = 279)	TGC>TAC	C469Y	0/128 (0)	0/84 (0)	1/67 (1.49)
	TAT>TGT	Y558C	0/128 (0)	0/84 (0)	1/67 (1.49)
	CGT>CAT	R561H	0/128 (0)	0/84 (0)	1/67 (1.49)
	GCT>GTT	A675V	0/128 (0)	1/84 (1.19)	0/67 (0)
*pfcrt* (*n* = 225)	ATG>ATT	M74I	19/116 (16.38)	7/64 (10.94)	6/45 (13.33)
	AAT>GAA	N75E	19/116 (16.38)	7/64 (10.94)	7/45 (15.56)
	AAA>ACA	K76T	19/116 (16.38)	7/64 (10.94)	7/45 (15.56)
*pfmdr1* (*n* = 235)	AAT>TAT	N86Y	6/116 (5.17)	1/51 (1.96)	1/68 (1.47)
	AGT>AAT	S163N	0/116 (0)	0/51 (0)	1/68 (1.47)
	TAT>TTT	Y184F	61/116 (52.59)	31/51 (60.78)	38/68 (55.88)
	ACT>TCT	T199S	1/116 (0.86)	0/51 (0)	0/68 (0)
	TCT>TTT	S1217F	1/116 (0.86)	0/51 (0)	0/68 (0)
	GAT>TAT	D1246Y	2/116 (1.72)	1/51 (1.96)	0/68 (0)

Notes: ^1^ Data are presented as the number of variant-carrying isolates/total isolates (variant frequency, %). ^2^ Percentages may not sum to 100.00 due to rounding.

**Table 4:** Haplotype composition of *pfk13*, *pfcrt* and *pfmdr1* genes in *P. falciparum* isolates imported from Africa.

Gene	Variant Pattern	Haplotypes	No. of Isolates	Proportion (%)
*pfk13* (*n* = 279)	Wild type	C_469_Y_558_R_561_A_675_	275	98.57
	Single variant	Y_469_Y_558_R_561_A_675_	1	0.36
	Single variant	C_469_C_558_R_561_A_675_	1	0.36
	Single variant	C_469_Y_558_H_561_A_675_	1	0.36
	Single variant	C_469_Y_558_R_561_V_675_	1	0.36
*pfcrt* (*n* = 225)	Wild type	M_74_N_75_K_76_	192	85.33
	Double variant	M_74_E_75_T_76_	1	0.44
	Triple variant	I_74_E_75_T_76_	32	14.22
*pfmdr1* (*n* = 235)	Wild type	N_86_S_163_Y_184_T_199_S_1217_D_1246_	102	43.40
	Single variant	Y_86_S_163_Y_184_T_199_S_1217_D_1246_	2	0.85
	Single variant	N_86_S_163_F_184_T_199_S_1217_D_1246_	122	51.91
	Single variant	N_86_S_163_Y_184_S_199_S_1217_D_1246_	1	0.43
	Double variant	Y_86_S_163_F_184_T_199_S_1217_D_1246_	3	1.28
	Double variant	N_86_N_163_F_184_T_199_S_1217_D_1246_	1	0.43
	Double variant	N_86_S_163_F_184_T_199_F_1217_D_1246_	1	0.43
	Triple variant	Y_86_S_163_F_184_T_199_S_1217_Y_1246_	3	1.28

Notes: ^1^ n, number of successfully sequenced isolates. ^2^ Percentages may not sum to 100.00 due to rounding.

## Data Availability

The data presented in this study are available on request from the corresponding author due to internal institutional restrictions on the release of raw parasite isolate sequencing datasets.

## Supplementary Materials

The following supporting information can be downloaded at: https://www.mdpi.com/article/10.3390/pathogens15101080/s1. Supplementary Table S1. Synonymous sites in *pfk13* and *pfmdr1* genes of *P. falciparum* isolates imported from Africa. No synonymous substitution was detected in *pfcrt*.

## Abbreviations

The following abbreviations are used in this manuscript:

ACT	artemisinin-based combination therapy
AL	artemether-lumefantrine
AS-AQ	artesunate-amodiaquine
DHA-PPQ	dihydroartemisinin-piperaquine
PCR	polymerase chain reaction
*P.**falciparum*	*Plasmodium falciparum*
*pfcrt*	*P. falciparum* chloroquine resistance transporter gene
*pfk13*	*P. falciparum kelch 13* gene
*pfmdr1*	*P. falciparum* multidrug resistance gene 1
WHO	World Health Organization
