# Supplementary material for: Molecular Surveillance of Antimalarial Drug Resistance-Associated Mutations in P. falciparum Imported from Africa into Guangdong Province, China, 2023–2025
Source: Pathogens. 2026 Oct 14;15(10):1080. doi: 10.3390/pathogens15101080 (PMC13708556; doi:10.3390/pathogens15101080)
Supplement: Supplementary file 1 [file pathogens-15-01080-s001.zip › pathogens-4573162-supplementary.pdf]

Supplementary Table S1. Synonymous sites in *pfk13* and *pfmdr1* genes of *P. falciparum* isolates imported from Africa. No synonymous substitution was detected in *pfcrt*.

Gene	Nucleotide mutation	Amino acid substitution	Variant-carrying isolates / total isolates (variant frequencies, %)		
			2023	2024	2025
pfk13 (n=279)	CGT>CGC	R471R	1/128(0.78)	0/84(0)	0/67(0)
	CGT>CGG	R513R	1/128(0.78)	0/84(0)	0/67(0)
	GGC>GGA	G548G	0/128(0)	0/84(0)	1/67(1.49)
	CCG>CCA	P553P	1/128(0.78)	0/84(0)	0/67(0)
	GCT>GCA	A621A	0/128(0)	1/84(1.19)	0/67(0)
pfmdr1 (n=235)	GGT>GGC	G102G	3/116(2.59)	4/51(7.84)	2/68(2.94)
	TCT>TCG	S149S	1/116(0.86)	0/51(0)	0/68(0)
	ACT>ACG	T1069T	7/116(6.03)	2/51(3.92)	5/68(7.35)
	TCA>TCC	S1137S	1/116(0.86)	0/51(0)	2/68(2.94)
	AAT>AAC	N1189N	1/116(0.86)	0/51(0)	0/68(0)
	AAT>AAC	N1227N	0/116(0)	1/51(1.96)	0/68(0)

Notes:

[1] Data are presented as the number of variant-carrying isolates / total isolates (variant frequency, %).

[2] Percentages may not sum to 100.00 due to rounding.
